# Study of Adverse Events Associated With the Use of Medical Devices Reported Under the Materiovigilance Programme of India in a Tertiary Care Teaching Hospital

**DOI:** 10.7759/cureus.101042

**Published:** 2026-01-07

**Authors:** Shruti Chandra, Umar Farooque Shaikh, Deepak Bhosle

**Affiliations:** 1 Clinical Pharmacology and Therapeutics, MGM Medical College and Hospital, Chhatrapati Sambhajinagar, MGM Institute of Health Sciences, Navi Mumbai, IND; 2 Clinical Research, MGM Medical College and Hospital, Chhatrapati Sambhajinagar, MGM Institute of Health Sciences, Navi Mumbai, IND

**Keywords:** materiovigilance, mdae, medical device adverse events, medical device risk classification 2017, mvpi, patient safety

## Abstract

Background

Medical device adverse events (MDAEs) are critical incidents in healthcare systems that can lead to patient harm, necessitating thorough investigation and management. This study aims to comprehensively analyse MDAE reports collected over six months at a Medical Device Management Centre (MDMC). The investigation focuses on various aspects, including gender distribution, age demographics, types of medical devices implicated, department-wise distribution within hospitals, risk classification according to regulatory standards, and subsequent corrective and preventive actions undertaken.

Methods

The study collected and analysed 80 spontaneous MDAE reports from the MDMC database. Data were meticulously extracted, categorised, and quantified to facilitate detailed analysis. Mean and standard deviation were calculated for quantitative variables, and proportions were calculated for categorical variables to elucidate patterns and trends in MDAE occurrences.

Results

Analysis revealed a gender disparity, with a higher proportion of MDAEs occurring in males (62%) compared to females (38%). Middle-aged individuals (41-50 years) constituted the largest age group affected by MDAEs, comprising 36.25% of reported cases. Patient monitors, IV catheters, and syringes emerged as the most frequently implicated medical devices, with patient monitors alone accounting for 18.75% of reported events. The Medicine ICU and Dialysis Unit reported the highest number of MDAEs, with 12.5% and 10% of total cases, respectively. Risk classification based on regulatory standards categorised the majority of events as Class B (51.25%), indicating low to moderate risk, while a notable proportion were classified as Class C (32.5%), signifying moderate to high risk. Additionally, in our study, most corrective actions after an MDAE involved repairing and returning the device for hospital use (94.59%). Two cases (5.41%) were sent to the manufacturer for replacement. Additionally, 43 out of 80 adverse events involved discarding single-use devices in hospital facilities. Preventive measures, such as staff training programs, were proposed to mitigate future risks.

Conclusion

This study highlights important patterns in patient safety risks. Risk classification demonstrated that most events fell within Class B and Class C categories. Overall, this study emphasises the importance of systematic reporting, analysis, and management of MDAEs to reduce patient harm. Strengthening preventive measures, enhancing staff awareness, and ensuring robust regulatory compliance are essential steps toward improving medical device safety in hospital settings.

## Introduction

As per the World Health Organisation (WHO), a medical device encompasses any tool, equipment, device, apparatus, implant, substance for in vitro utilisation, software, substance, or any other analogous or linked item intended by the manufacturer for utilisation, either independently or together, for medical objectives [1].

Medical devices are integral to the healthcare delivery system and contribute significantly to patient care. Medical devices can diagnose, treat, and prevent numerous diseases. They differ from simple lifesaving devices, such as thermometers or tongue depressors, to complicated implants, such as heart valves or cardiovascular stents [2].

As per the regulations outlined in the Indian Medical Device Rule (MDR) 2017, medical devices refer to instruments designed for use either internally or externally to diagnose, treat, alleviate, or prevent diseases or disorders in humans or animals that are periodically notified under the Drug and Cosmetics Act (DCA), including mechanical contraceptives (intrauterine devices, condoms, tubal rings), disinfectants, and insecticides notified by the government under the DCA, surgical devices, and other items [3].

A medical device adverse event (MDAE) is an unpredicted or unsuitable medical event, accidental illness, injury, or discomfort in participants [4].

The Materiovigilance Programme has been approved by the Ministry of Health and Family Welfare (MoHFW), Government of India, and the Drug Controller General of India (DCGI) on July 6, 2015, at the Indian Pharmacopoeia Commission (IPC) in Ghaziabad to address the potential adverse events associated with medical devices. Materiovigilance can be defined as the vigilant surveillance of any adverse occurrences arising from the use of medical devices, facilitated by a comprehensive system encompassing identification, collection, reporting, evaluation of adverse events, and subsequent implementation of safety measures or corrective actions during the post-marketing phase [1]. From the start of the Materiovigilance Programme from July 2015 to October 2019, the IPC received and analysed more than 1931 MDAEs, 1277 (66.1%) of which were serious [4]. In 2016, the global market for medical devices was predicted to be worth 380 billion United States dollars (USD), up from 260 billion USD in 2006. India is one of the top 20 medical device businesses in the world, contributing $3 billion in USD to the industry. However, there is no appropriate mechanism in India for recording MDAEs or maintaining medical device safety records [5].

Before the MDR 2017, all medical devices were regulated by the DCA of 1940. In the MDR 2017, medical devices and drugs were separated for regulatory purposes. The MDRs of 2017 classify medical devices based on their risk level. Class A devices are low risk, such as thermometers, surgical dressings, and swabs. Class B devices are low to moderate risk, including hypodermic needles and suction equipment. Class C devices are moderate to high risk, such as ventilators, intraocular lenses, and bone fixation plates. Class D devices are high risk, including cochlear implants, implantable defibrillators, and cardiac stents.

The Materiovigilance Programme of India (MvPI) aims to collect data on medical device-related adverse events systematically and scientifically analyse them to aid in regulatory decisions and recommendations on the safe use of medical devices being made using data generated from India [6]. Also, monitor MDAE, create awareness among healthcare professionals about the importance of MDAE reporting in India, and monitor the benefit-risk profile of medical devices [7]. The Mission of MvPI is to safeguard the health of the Indian population by ensuring that the benefit of the use of Medical Devices outweighs the risk associated with its use [8]. The Vision of MvPI is to improve patient safety and welfare by monitoring adverse events related to medical devices, thereby reducing the risk associated with the use of medical devices [9].

Therefore, we planned this study as MDAEs often go unrecognised and underreported by healthcare professionals and the public. The present study aims to increase awareness about the importance of reporting adverse events by providing information on Materiovigilance.

## Materials and methods

The observational study is a part of the MvPI. It presents the data collected from the Medical Device Adverse Event Monitoring Centre (MDMC) under MvPI at MGM Medical College and Hospital, Aurangabad. The study commenced after the Ethics Committee approved it for six months (EC Approval Number: MGM-ECRHS/2023/104). The MDAE reporting form, recommended by the IPC Ghaziabad, has been used as a data collection tool [10]. We employed active and passive surveillance for data collection. In the Active Surveillance Study, data were collected by visiting various medical and surgical departments. We went to various departments to collect the MDAE actively. The collection and reporting of MDAEs were performed in accordance with the relevant guidelines and regulations given by the MvPI. In passive surveillance, we conducted a targeted awareness program for healthcare professionals on MvPI and how to report MDAEs in a prescribed form.

Statistical analysis

Data was entered in Microsoft Excel 2021 (Microsoft Corp., Redmond, WA) and analysed. Mean and standard deviation were calculated for quantitative variables, and proportions were calculated for categorical variables. Data were represented in the form of visual impressions like bar diagrams, graphs, and tables.

## Results

**Figure 1:**
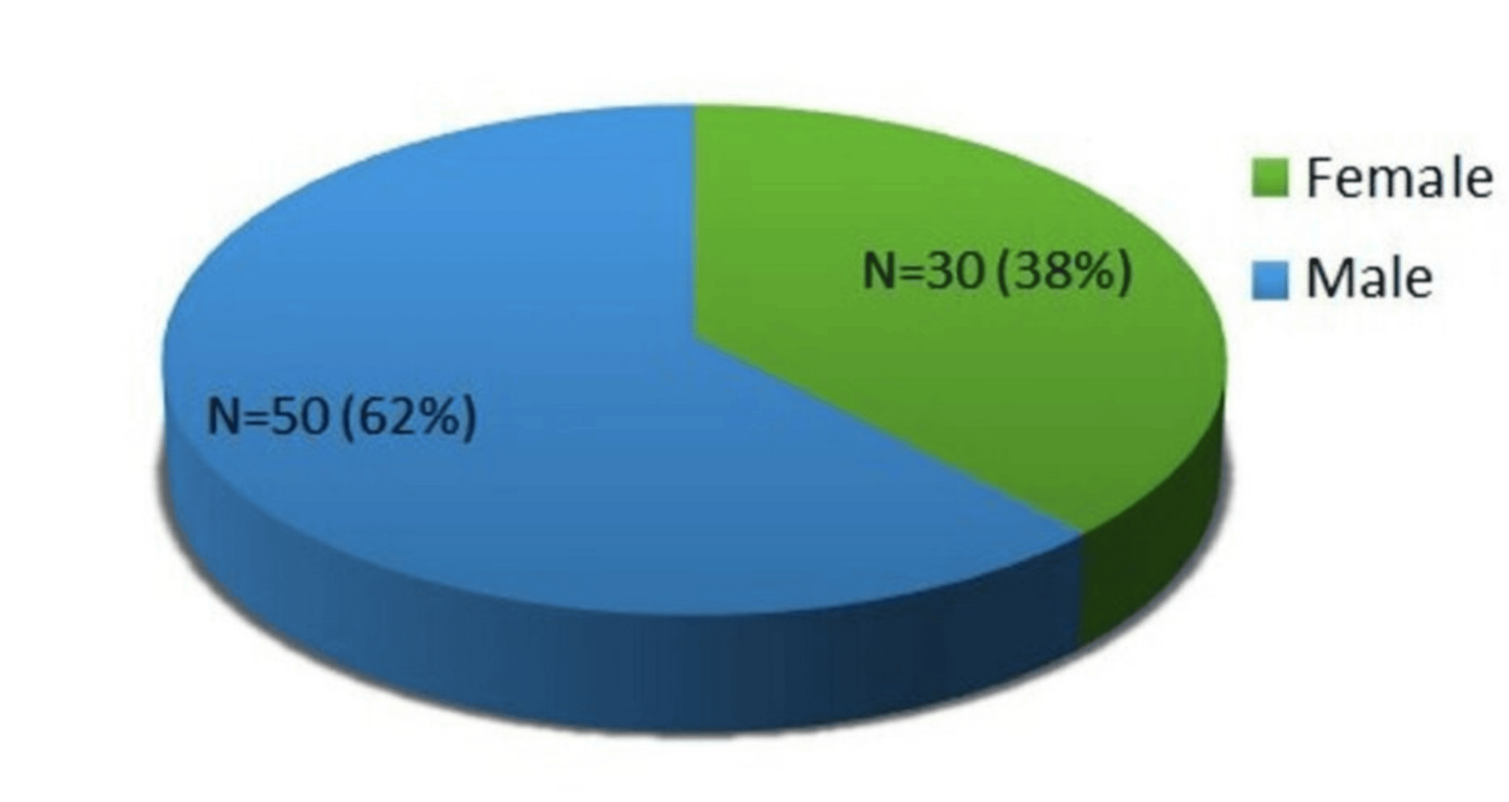
Pie diagram to show sex-wise distribution of MDAE in patients MDAE, medical device adverse event

During a span of six months at the MDMC, a total of 80 spontaneous MDAE reports were collected and analysed. Among these reports, MDAE occurrences were more dominant in males, accounting for 50 cases (62%), while in females, the occurrences were 30 cases (38%), totalling 80 patients, as shown in Figure 1. Data collected by using the MDAE reporting form [10].

**Figure 2:**
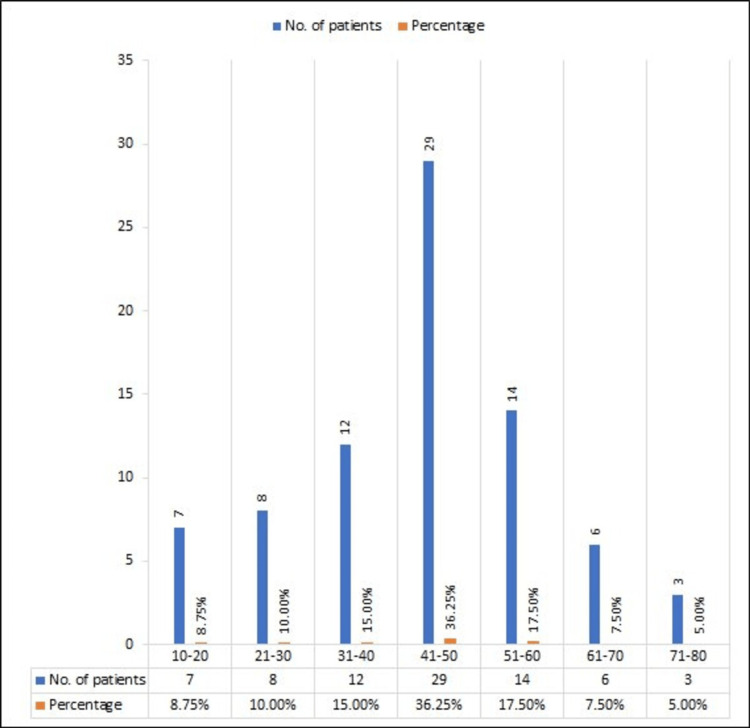
Data distribution based on age

Figure 2 shows data distribution based on age collected by using the MDAE reporting form [10]. In the respective age groups, there were seven patients between 10 and 20 years old, accounting for 8.75% of the total. In the age group from 21 to 30 years old, there are eight patients, accounting for 10.00%. In the age group from 30- to 40-year-old group, there are 12 patients, accounting for 15.00%. The age group from 41 to 50 has 29 patients, accounting for 17. 50%. In the age group from 51 to 60, there are 14 patients, accounting for 17. 50% of the total number of patients. In the age group 61-70, there are six patients, accounting for 7.50%. Finally, the age group 71-80 has three patients, accounting for 5.00%. The mean age is 43.87, and the standard deviation is ±14.19.

The data represented in Table 1 were derived from the MDAE reporting form [10]. In six month study period, we received 15 MDAE of BP monitor (18.75%), eight MDAEs of IV set (10%), four MDAE associated with syringes (5.00%), four MDAE related to a syringe pump (5%), three MDAE due to defibrillators, glucose monitors, haemodialysis machines, nebulizers and respiratory humidifiers, and digital thermometers (3.75%), two MDAEs caused by dialyser, electrocardiographs, surgical tape, nebulizer masks, and surgical dressing kits (2.50%), one (1.25%) MDAE related to arterial blood pressure (ABP), blood collection needles, binocular microscopes, dressing gauze, biopsy guns, skin markers, central venous catheters, Foley balloon catheters, high-concentration oxygen masks, infant feeding tubing, IV cannula fixators, nasal cannulas, orthopedic heating bags, pressure monitoring lines, spirometers, suction catheters, bandages, ventilators, and ventilator circuit plans.

**Table 1:** Medical device-wise total numbers of reported ADRs ADR, adverse drug reaction

Medical Device Name	Total Number of Reported ADRs	Percentage
Patient Monitor	15	18.75
IV Catheter	8	10
Syringe	4	5
Syringe Pump	4	5
Defibrillator	3	3.75
Glucose Monitor	3	3.75
Haemodialysis Machine	3	3.75
Nebulizer	3	3.75
Respiratory Humidifier	3	3.75
Digital Thermometer	3	3.75
Electrocardiograph	2	2.5
Surgical Tape	2	2.5
Nebulizer Mask	2	2.5
Surgical Dressing Kit	2	2.5
Nasal Cannula	2	2.5
ABPM	1	1.25
Blood Collection Needle	1	1.25
Binocular Microscope	1	1.25
Dressing Gauze	1	1.25
Biopsy Gun	1	1.25
Skin Marker	1	1.25
Dialyzer	1	1.25
Central Venous Catheter	1	1.25
Foley Balloon Catheter	1	1.25
High-Concentration Oxygen Mask	1	1.25
Infant Feeding Tubing	1	1.25

Table 2 depicts that the Medicine ICU reported 10 cases (12.5%), followed by the dialysis unit with eight cases (10%). The emergency ICU, medicine ward, and surgical ICU each reported six cases (7.5%), while the pulmonary ward reported four cases (5%). Additionally, the cardiac ICU, Medical Centre and Research Institute (MCRI) ICU, nephrology ward, and psychiatric ward each reported three cases (3.75%). The ENT ward, Isolation ICU, Kidney Transplant ICU, Medicine OPD, Obstetrics and Gynaecology ICU, Orthopaedic Ward, Paediatric ICU, Paediatric Ward, and Super Deluxe Ward each reported two cases (2.5%). Finally, one case (1.25%) of MDAE was reported from the Blood Collection OPD, Central Pathology Laboratory, Deluxe Ward, Dermatology OPD, Ophthalmology Ward, Surgery OPD, Surgery Ward, Surgical OPD, Surgical Operation Theatre, and Urology Ward. These data were collected by using the MDAE reporting form [10].

**Table 2:** MDAEs reported from different departments of hospitals MDAE, medical device adverse event

Name of Department	Total Number of MDAE Reported	Percentage
Medicine ICU	10	12.5
Dialysis Unit	8	10
Emergency ICU	6	7.5
Medicine ward	6	7.5
Surgical ICU	6	7.5
Pulmonary Ward	4	5
Cardiac ICU	3	3.75
MCRI ICU	3	3.75
Nephrology Ward	3	3.75
Psychiatric Ward	3	3.75
ENT Ward	2	2.5
Isolation ICU	2	2.5
Kidney Transplant ICU	2	2.5
Medicine OPD	2	2.5
Obstetrics and Gynaecology ICU	2	2.5
Orthopaedic Ward	2	2.5
Paediatric ICU	2	2.5
Paediatric Ward	2	2.5
Super Deluxe Ward	2	2.5
Blood Collection OPD	1	1.25
Central Pathology Laboratory	1	1.25
Deluxe Ward	1	1.25
Dermatology OPD	1	1.25
Ophthalmology Ward	1	1.25
Surgery OPD	1	1.25
Surgery Ward	1	1.25
Surgical OPD	1	1.25
Surgical Operation Theatre	1	1.25
Urology Ward	1	1.25
Total	80	100

**Figure 3:**
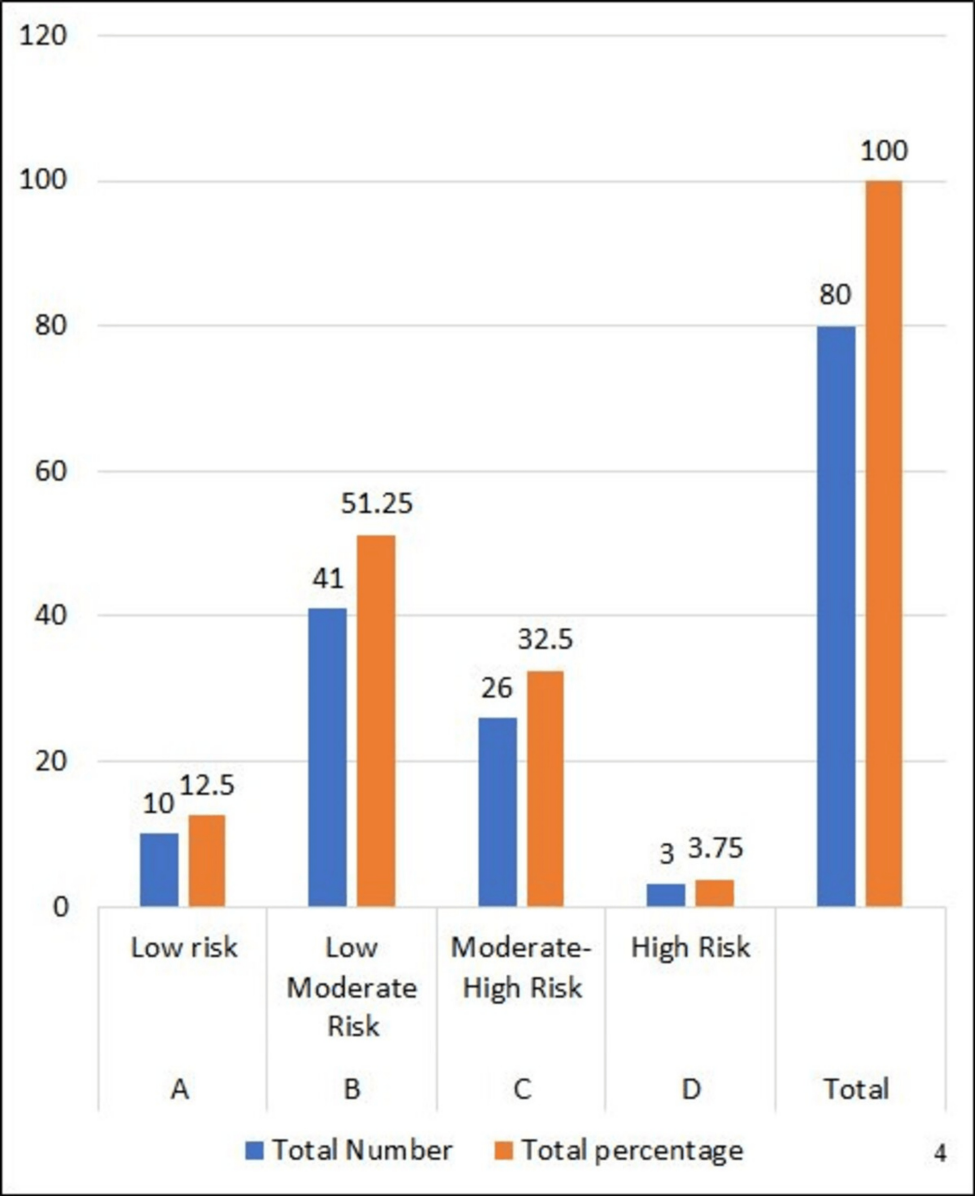
Device risk classification as per Medical Device Rule 2017 Reference: [4]

In total, we analysed 80 adverse events associated with medical devices in our study. According to the guidelines outlined in the MDR of 2019 [4], our study investigated device risk classification depicted by Figure 3. We identified eight cases, constituting 10% of the total, classified as Class A, denoting low risk. The most prevalent were Class B events, categorised as Low Moderate Risk, accounting for 51.27% of the total with 41 cases. Class C events, indicating Moderate-High Risk, were observed in 26 cases, representing 32.5% of the total. Finally, there were three cases, making up 3.75% of the total, classified as Class D, signifying High Risk. Data was collected by using the MDAE reporting form, which indicates risk classification [10].

**Figure 4:**
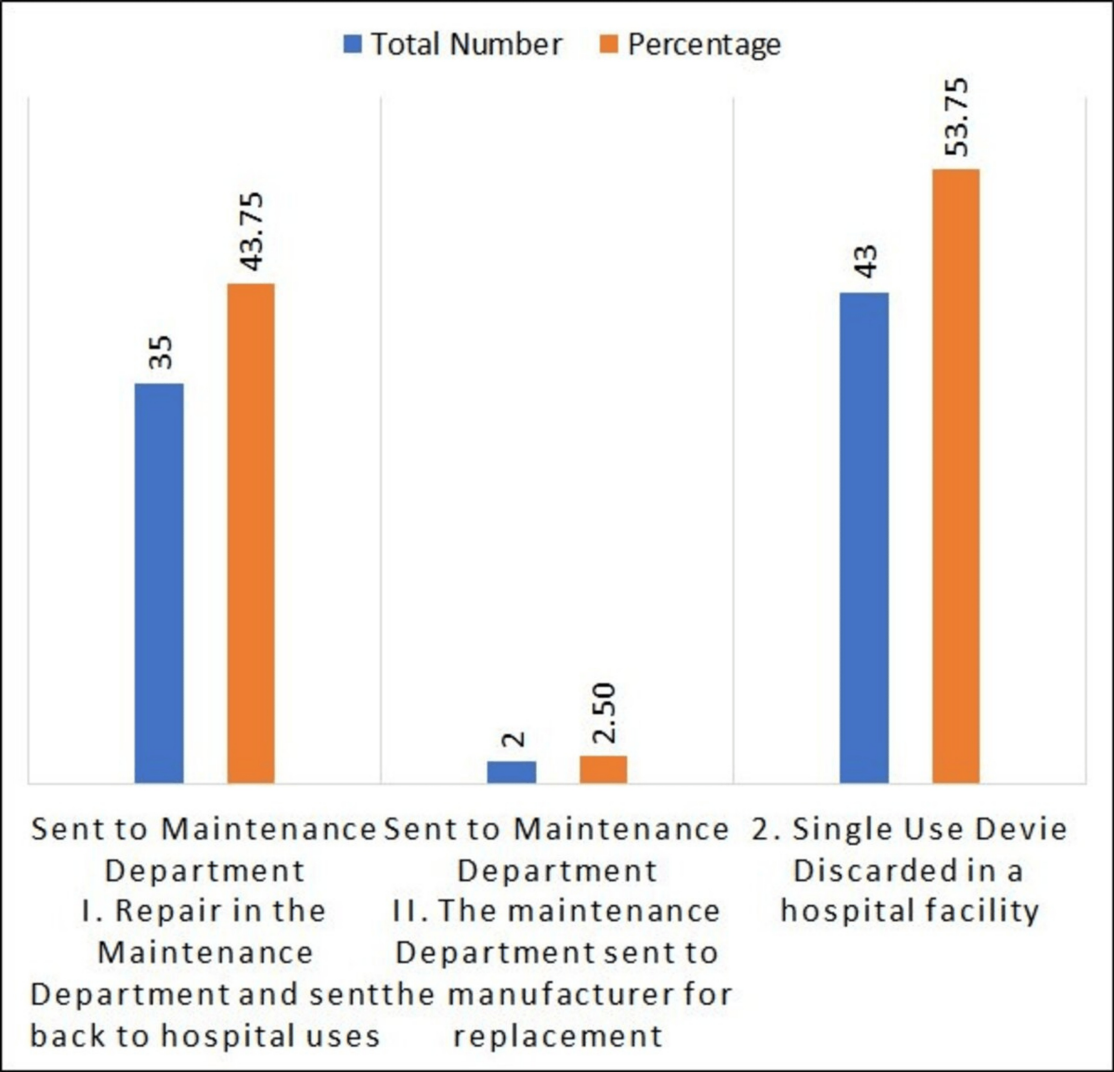
List of corrective actions

Figure 4 shows a list of corrective actions after MDAE occurrences. For example, a total of 37 were sent to the Maintenance Department, out of which 35 (43.7%) underwent repair in the Maintenance Department and were sent back to hospital use, while two (2.5%) instruments were sent back to the manufacturer for replacement. Forty-three (53.7%) were single-use devices, discarded in a hospital facility.

Table 3 showed that, in our study, we diligently reported a total of 80 MDAEs to the National Coordination Centre-Materiovigilance Programme of India (NCC-MvPI) and the IPC using the MDAE reporting form [10]. From these reports, we received acknowledgements detailing various issues. Device malfunction was identified as the cause in 41 cases (51.25%) of MDAEs, while poor quality was cited in 29 cases (36.25%). Additionally, blockage was reported in four cases (5.00%), reading errors in three cases (3.75%), and itching, leakage, and redness each in one case (1.25%). This comprehensive reporting underscores our commitment to ensuring the safety and efficacy of medical devices used in healthcare settings.

**Table 3:** Adverse event acknowledged by NCC-MvPi, IPC after reporting MDAEs MDAE, medical device adverse event; NCC-MvPi, National Coordination Centre-Materiovigilance Programme of India

Adverse Event Acknowledged by NCC-MvPi, IPC, by Type	Total Number	Total Percentage
Device Malfunction	41	51.25
Poor Quality	29	36.25
Blockage	4	5
Reading Error	3	3.75
Itching	1	1.25
Leakage	1	1.25
Redness	1	1.25
Total	80	100

## Discussion

Medical devices have been crucial in diagnosing and treating a range of illnesses [11]. With the progress that has been made in technology and the increasing popularity of high-quality health care around the world, the medical devices sector is estimated to generate more than USD 350 billion in annual revenue. In the healthcare sector, India saw a growth rate of 20%. These devices have also created substantial risks to patients with high-profile recalls [12]. US FDA Defined MDAE is an adverse event that can refer to any unfavourable occurrence involving medical devices in the market that resulted in or contributed to a fatality or severe harm, or experienced a malfunction that has the potential to result in or contribute to a fatality or severe injury if the malfunction were to happen again [13].

In 2003, there were 366 reports of adverse reactions to medical devices, increasing to 6,101 in 2006, 12,374 in 2007, 40,940 in 2008, and 53,304 in 2009. In 2012, more than 180,000 suspicious MDAEs were reported, of which 13% resulted in serious injury and 0.06% led to death. Most (71%) of the MDAEs cases were reported by medical institutions, while 22% were reported by distributors, 3% were reported by manufacturers, and 5% were reported by individuals [14]. Therefore, the Materiovigilance Programme was started with the intention to collect safety information systematically, to monitor adverse drug reactions associated with medical devices, to raise awareness among healthcare professionals about the importance of reporting adverse drug reactions in India, and to monitor the benefit-risk profile of medical devices. Based on these reports, a decision will be taken on whether any change is required in the labelling of the devices, or whether a recall or a ban [15] is required.

The current study aimed to investigate adverse events associated with the use of medical devices reported under the Materiovigilance Programme of India in a tertiary care hospital over six months. In our study, patient monitors accounted for the highest number of reported MDAEs, with 15 cases (18.75%) experiencing adverse events associated with device malfunction. This was followed by IV catheters, with eight cases (10%) reporting adverse events related to poor quality. Additionally, syringes were associated with four cases (5%) of reported MDAEs, with adverse events also attributed to poor quality. These findings align with those of a study conducted by Najmi et al. in 2022 [16], which reported similar frequencies of adverse events based on medical devices, with BP instruments accounting for 16 cases (5%). Our investigation observed the incidence of MDAEs across various departments. The Medicine ICU recorded 10 cases (12.5%), followed by the dialysis unit with eight cases (10%). Similarly, the emergency ICU, medicine ward, and surgical ICU each reported six cases (7.5%), while the pulmonary ward documented four cases (5%). Additionally, the cardiac ICU, MCRI ICU, nephrology ward, and psychiatric ward individually reported three cases (3.75%). The ENT ward, Isolation ICU, Kidney Transplant ICU, Medicine OPD, Obstetrics and Gynaecology ICU, Orthopaedic Ward, Paediatric ICU, Paediatric Ward, and Super Deluxe Ward each registered two cases (2.5%). Singular cases (1.25%) of MDAE were reported from the Blood Collection OPD, Central Pathology Laboratory, Deluxe Ward, Dermatology OPD, Ophthalmology Ward, Surgery OPD, Surgery Ward, Surgical OPD, Surgical Operation Theatre, and Urology Ward. A similar trend was noted in the study by Samal et al. in 2022 [17], conducted over 15 months, where 56 MDAEs were reported from different hospital departments. Medicine reported 16 cases (28.6%), OPD recorded nine cases (16.1%), outside data had eight cases (14.3%), the laboratory reported five cases (8.9%), and surgery documented four cases (7.1%). Moreover, ophthalmology and outside hospitals each reported three cases (5.4%), while casualty and dental departments each recorded two cases (3.6%). Obstetrics and Gynaecology, ICU, and Paediatrics each reported one case (1.8%).

In our study, we found that the majority of corrective actions taken after an MDAE involved repairing the device in the maintenance department and returning it for hospital use. This accounted for 35 cases (94.59%). Additionally, the maintenance department sent two cases (5.41%) to the manufacturer for replacement. Another significant corrective action, numbered two, was the discarding of single-use devices in a hospital facility, which occurred in 43 out of 80 MDAEs.

In our investigation, the class-wise distribution of MDAEs was as follows: Class A (low risk), 10 (12.5%); Class B (low to moderate risk), 41 (51.25%); Class C (moderate to high risk), 26 (32.5%); and Class D (high risk), three (3.75%).

In contrast to our study, PK et al. [18] conducted a three-year study and reported the following class-wise distribution of MDAEs: Class A (low risk), five (2%); Class B (low to moderate risk), 161 (66%); Class C (moderate to high risk), 59 (24%); and Class D (high risk), 20 (8%), for a total of 224 MDAEs.

In a study done in San Francisco, over 35 months, the result of the study was that out of 517 sudden deaths observed, 22 individuals (4.3%) had cardiac implantable electronic devices (CIEDs) [19]. A study conducted by Machado-Alba et al., a three-year retrospective study, reported that the most frequently reported adverse events belonged to the vascular access category, accounting for 738 (45.2%) cases, followed by surgical equipment with 330 (20.2%), airway devices with 242 (14.8%), biosafety equipment with 96 (5.9%), urinary or digestive catheters with 60 (3.7%), vital signs monitoring equipment with 56 (3.4%), healing materials with 39 (2.4%), and drainage bags with 38 (2.3%) [20].

## Conclusions

This study offers only a glimpse into the broader landscape of reporting trends for such incidents in tertiary care hospitals, indicating that it represents merely a fraction of the overall picture. Disparities exist in reporting among healthcare professionals of varying grades and across different departments, underscoring the importance of regularly conducting awareness initiatives, training sessions, and workshops. Through consistent and comprehensive reporting, decisions can be made regarding the necessity for changes in device labelling or the possibility of recalling or banning specific products. Therefore, there is a critical need to prioritise the safety of medical devices and enhance reporting mechanisms through further research and attention.
